# Dynamic analysis of QTLs on plant height with single segment substitution lines in rice

**DOI:** 10.1038/s41598-022-09536-8

**Published:** 2022-03-31

**Authors:** Yu Fu, Hongyuan Zhao, Jiongkai Huang, Haitao Zhu, Xin Luan, Suhong Bu, Zupei Liu, Xiaoling Wang, Zhiqin Peng, Lijun Meng, Guifu Liu, Guiquan Zhang, Shaokui Wang

**Affiliations:** 1 Guangdong Key Laboratory of Plant Molecular Breeding, South China Agricultural University, Guangzhou, 510642 People’s Republic of China; 2 National Engineering Research Center of Rice (Nanchang), Rice Research Institute, Jiangxi Academy of Agricultural Sciences, Nanchang, 330299 People’s Republic of China; 3 Agricultural Genomics Institute in Shenzhen, Chinese Academy of Agricultural Sciences, Shenzhen, 440307 People’s Republic of China

**Keywords:** Genetics, Development, Genetic interaction, Quantitative trait

## Abstract

Dynamic regulation of QTLs remains mysterious. Single segment substitution lines (SSSLs) and conditional QTL mapping and functional QTL mappings are ideal materials and methods to explore dynamics of QTLs for complex traits. This paper analyzed the dynamics of QTLs on plant height with SSSLs in rice. Five SSSLs were verified with plant height QTLs first. All five QTLs had significant positive effects at one or more developmental stages except QTL_1_. They interacted each other, with negative effects before 49 d after transplanting and positive effects since then. The five QTLs selectively expressed in specific periods, mainly in the periods from 35 to 42 d and from 49 to 56 d after transplanting. Expressions of epistasis were dispersedly in various periods, negative effects appearing mainly before 35 d. The five QTLs brought the inflexion point ahead of schedule, accelerated growth and degradation, and changed the peak plant height, while their interactions had the opposite effects. The information will be helpful to understand the genetic mechanism for developmental traits.

## Introduction

Plant type of rice, including root type, stem type, leaf type and spike type, is one of the main factors determining yield; thus, shaping the ideotype is an important way to improve rice yield^1–3^. Plant height is a crucial component of plant type traits. On the one hand, plant height is closely related to lodging resistance, and plant lodging during maturation surely results in a sharp decline in the yield and quality of rice^4^. The development of dwarf and semidwarf rice cultivars has greatly increased the capacity of lodging resistance and the potential yield since the 1950s. On the other hand, plant height is a major determinant of a plant’s ability to compete for light because of its close correlation with leaf number and leaf distribution; high stems being usually accompanied by high biomass and then high grain yield^5,6^. This conflicting ideotype suggests that a suitable plant height should be retained between 90 and 120 cm to obtain the optimal output in cereal crops^7^. Understanding the genetic basis of plant height therefore makes it possible to find a balance between high yield and lodging. Specifically, plant height is a relatively easily investigated trait that can be measured at a series of stages to allow us to dynamically explore the genetic mechanism of development. Thus, plant height is often used as a model trait for the study of developmental behaviors^8–10^.

QTL mapping is an effective approach to explore the genetic mechanisms of quantitative traits. For developmental traits such as plant height, tiller number and leaf number, common QTL mapping methods can be summarized as (1) unconditional mapping, (2) conditional mapping and (3) functional mapping^11^. Unconditional QTL mapping usually directly analyzes the phenotypic values measured at various growth stages and then infers the dynamic genetic architecture of a developmental trait by longitudinal comparison of the mapping results^12^. Conditional QTL mapping needs to first estimate the conditional effects $y(t|t - 1)$ for the phenotypic values at time $(t)$ given the phenotypes at time $(t - 1)$^13,14^ and then to conduct QTL mapping based on these estimations^8,9,15,16^. Conditional QTL mapping can effectively measure the net expression of genes from time $(t - 1)$ to time $(t)$ since $y(t|t - 1)$ is independent of $y(t - 1)$ (the phenotypic value at time $(t - 1)$)^17^. Functional QTL mapping includes three steps: fitting a mathematical model for the development of a trait, estimating the parameters in the model that defined the function, and then QTL mapping for these parameters^18–21^. The advantage of functional QTL mapping is to detect QTLs that regulate the shape and trajectory of trait changes owing to the incorporation of biological principles^22^. Studies using these strategies have provided a wealth of information about QTLs controlling the developmental behavior of traits, such as QTL accumulated effects at a point in time, QTL net effects in a period of time, and functional QTLs changing the process of development. Over the past 30 years, great efforts have been made to dissect the genetic basis of plant height^8–10,16,23–25^. Studies have indicated that plant height in rice is generally controlled by both qualitative and quantitative genes, more than 1000 QTLs have been mapped on rice chromosomes, and their action mechanisms manifest mainly accumulation and interaction of QTL effects, i.e., additive effect and dominant or epistatic effect with the features of spatio-temporal expression (http://www.gramene.org/qtl).

However, most of the research materials applied in previous studies were conventional mapping populations such as F_2_ self-pollinating populations (F_2_), back-crossing populations (BC), double haploid lines (DHLs), and recombinant inbred lines (RILs), in which inconsistent genetic backgrounds among individuals or lines could disturb the analysis results^26^. Moreover, systematic studies on developmental traits have rarely been reported using various approaches simultaneously, especially those lacking to tail after the expression pattern for an identified QTL. In this paper, we applied five single segment substitution lines (SSSLs) as experimental materials to first identify whether putative QTLs were carried out on plant height for each SSSL, to then estimate conditional effects $y(t|t - 1)$ and four functional parameters in Wang-Lan-Ding’s model and to finally carry out unconditional, conditional and functional QTL mapping. Here, the conditional effects were estimated based on phenotypic values of plant height measured at nine time points of development. Wang-Lan-Ding’s model is about the second-order ordinary differential equation of insect developmental rate with respect to temperature^27^ (Wang et al. 1982). The model, similar to the well-known logistic model, was verified to be able to more accurately describe the entire developmental process of insects^28^ and can also be applied to fit the growth curve of developmental traits in crops^29^. We aimed to provide the first quantification of genetic patterns affecting plant height, including QTL effects and interactions, accumulated effects and net effects, as well as how functionally to regulate the growth curve of plant height, and a comprehensive understanding of dynamic genetic mechanisms for developmental traits such as plant height.

## Materials and methods

### Materials and mapping population

**Figure 1:**
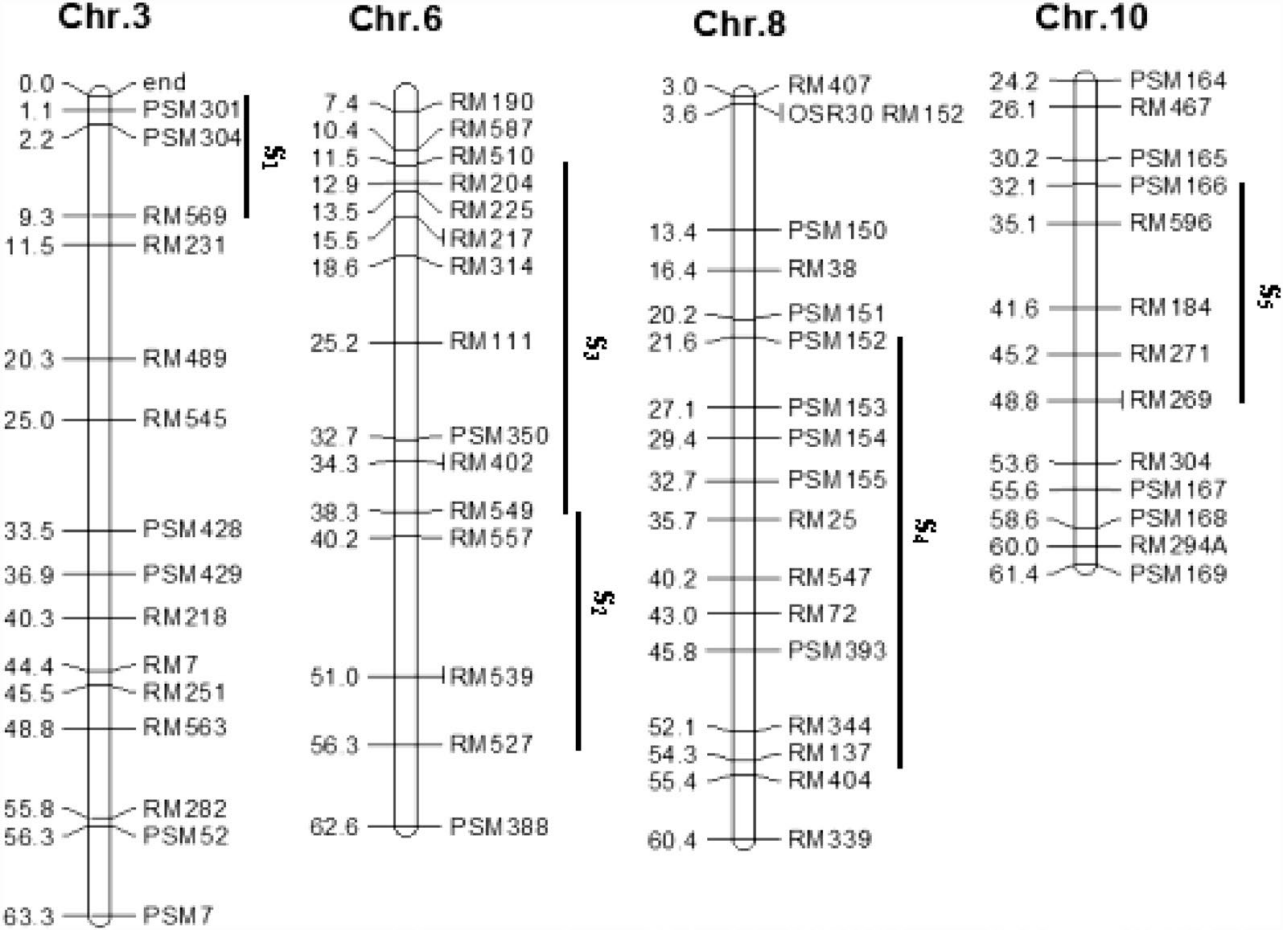
The approximate lengths and locations of substitution segments on chromosomes. Chr is the abbreviation of chromosome, which was followed by chromosomal number. Genetic distance (cM) and marker codes are listed on the left and right of Chr, respectively. The vertical lines on the right of Chr represent substitution segments with serial numbers $S_{i}$

Similar experimental materials as a previous study^30^, Huajingxian 74 (HJX74) and its five single segment substitution lines (SSSLs) (Table 1), were applied in this trial. HJX74 is an elite *indica* variety with many excellent properties cultured by our laboratory in South China. Each SSSL possessed only a single substituted segment from a donor under the HJX74 genetic background and was distributed in related molecular marker regions on corresponding chromosomes with given lengths (Fig. 1). Double segment substitution lines (DSSLs) were polymerized based on the F_2_ populations from the crossing combinations of two SSSLs. A half diallel mating scheme was conducted using HJX74 and their SSSLs, DSSLs as crossing parents to generate the mapping population that included HJX74, 5 SSSLs, 7 DSSLs and 26 crossing combinations. Some crossing combinations were lacking since the seeds of F_1_ were scarce.

**Table 1:** Single-segment substitution lines (SSSLs) and their basic information.

SSSL	Code	Chr	Marker on subsitituted segment	Donor parent
W23-03-08-09-27-82	S_1_	3	End–PSM301–PSM304–RM569	Lemont
W08-18-09-09-06-02	S_2_	6	RM549–RM136–RM527	IR64
W04-47-68-05-04-04-02-02	S_3_	6	RM510–RM204–RM50–RM549	BG367
W06-26-35-01-05-02	S_4_	8	PSM152–PSM154-RM72–RM404	Katy
W11-17-03-07-05-08	S_5_	10	PSM166–RM596-RM271–RM269	Basmati 370

SSSL is the abbreviation of the single segment substitution line. S_i_ represented the code of *i*th SSSL. Chr was the abbreviation of chromosome.

### Field experiments and measurement of plant height

The same phenotypic trial as the previous study^30^ was applied in this study. The trial site was located at the teaching and experimental station of South China Agricultural University in Guangzhou, China (23° 79ʹ N, 113° 159ʹ E). In the early season (duration from March to July) of 2018, 39 genotypic materials were grown in a completely randomized block design with three replications. Germinated seeds were sown in a seedling bed, and then seedlings were transplanted to a rice field 20 days later, with one plant per hill and a density of 16.7 cm × 16.7 cm. Each plot consisted of four rows with ten plants per row. Local standard practices were used for the management of the trial. Plant height per hill on 10 central plants, the distance between the base and the top of the main stem of the plant, was measured in each plot from seven days after transplanting onwards, and data every 7 days once were continuously recorded for nine weeks (denoted by $t_{1}$ to $t_{9}$). The averages of plant height in each plot for the nine stages were used as input data for the subsequent analysis.

### The Wang–Lan–Ding mathematical model

The phenotypic performance $(y)$ of each plot during the nine measurement times $(t)$ can be described by the equation^27^:

$$ y = \frac{K}{{1 + \exp {(} - r{(}t - t_{0} {))}}} \times {(}1 - \exp {(} - \frac{{t - t_{\min } }}{c}{))} \times {(}1 - \exp {(} - \frac{{t_{\max } - t}}{c}{))} $$

In the above model, the first term is a logistic model in which the parameter $K$ is the upper limit of plant height, namely the potential maximum of plant height. $t_{0}$ is the inflexion point of the logistic curve, or the optimum time. $r$ is the growth rate, and $c$ is the degradation rate. The DUD (do not use derivatives) method was used to estimate all parameters in the model of the $(t_{1} ,y_{1} ),(t_{2} ,y_{2} ), \ldots ,(t_{9} ,y_{9} )$ curve for each plot using SAS software v9.13. Then, functional QTL mapping was conducted based on the estimations of the four parameters.

### Statistical analysis and estimation of QTL effects

The model $y_{ij} = \mu + G_{i} + B_{j} + e_{ij}$ was adopted to analyze the variances on the phenotypic performances $(y)$ of plant height measured for each plot at various stages, where $\mu , \, G, \, B$ and $e$ were the estimations of the population mean, genotype, block and residual error effect, respectively. *i* and *j* represent serial numbers of genotypes and blocks, respectively. The additive effect (*a*) or dominant effect (*d*) of QTLs was calculated by the estimations of $(S_{i} - HJX74)$, and the epistatic effect (*e*) between QTL pairs was calculated by $(D_{ij} + HJX74 - S_{i} - S_{j} ),$ where *S*, *D* and HJX74 indicate the phenotypic means of single-, dual-segment substitution materials and HJX74, respectively, and $i, \, j$ represents two homozygotes or heterozygotes of SSSLs.

Conditional variable $y_{t|t - 1}$ was estimated by the formula of $y_{t|t - 1} = y_{t} - b_{t/t - 1} (y_{t - 1} - \overline{y}_{t - 1} )$, presenting phenotypic value at time* t* conditional on the phenotypic value at given time *t* − 1*,* where $y_{t - 1} , \overline{y}_{t - 1}$ and $y_{t} , \, \mathop{y}\limits^{\rightharpoonup} _{t}$ were the phenotypic values and the means at times *t* − 1 and *t*, respectively. $b_{t/t - 1}$ is the regression coefficient for phenotypic values at time *t* versus those at time *t* − 1. QTL mapping was imposed on the conditional variable $y_{t|t - 1}$ to generate conditional QTLs. Statistical analysis and estimation of QTL effects were carried out with aov ( ) and lm ( ) functions in R language (https://www.r-project.org/).

The study was in compliance with relevant institutional, national, and international guidelines and legislation.

## Results

### Unconditional QTL mapping on plant height

**Figure 2:**
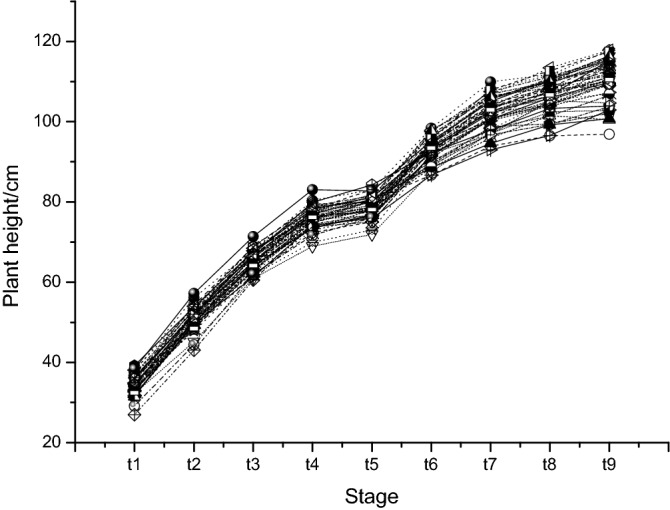
Growth curves of 39 genotypes for plant height over time. *ti* represents the *i*th stage measured, with an interval of 7 days. Unit of plant height was in cm.

Plant height approximately approached the “S” type of growth curve (Fig. 2). The figure drawn from all 39 genotypic materials indicated that after slow growth, plant height reached its peak and then started to decrease slightly. Separate analysis of variances at various stages revealed that a significant difference in plant height existed among genotypes (Supplementary Table 1), supporting the existence of QTLs for plant height in the mapping population. The contrast tests found that each of the five SSSLs harbored plant height QTLs (Table 2). All carried with additive and/or dominant effects detected at one or more stages (see $QTL(t)$ in Table 2). QTL on SSSL S_5_ (denoted by QTL_5,_ similarly hereinafter) detected with significant dominant effects just at one of stages perhaps was unreliable. The other QTLs repeatedly appeared guaranteed their truth. Only QTL_1_ exhibited negative effects, and the others increased plant height. During the early period (from $t_{0}$ to $t_{3}$), few QTLs were detected, while more QTLs were present in the middle-late period. The variations in QTL effects with time implied the dynamics of expression for these QTLs.

**Table 2:** Additive and dominant effects of SSSLs on plant height at various developmental stages compared with HJX74.

SSSL	Effect	QTL	*t*_*1*_	*t*_*2*_	*t*_*3*_	*t*_*4*_	*t*_*5*_	*t*_*6*_	*t*_*7*_	*t*_*8*_	*t*_*9*_
S_1_	*a*	QTL(*t*)						− 14.85*	− 6.63**	− 9.53**	− 12.97**
		QTL(*t|t* − 1)						− 6.57**		− 3.75**	
	*d*	QTL(*t*)								− 6.55**	− 7.71**
		QTL(*t|t* − 1)								− 4.96**	
S_2_	*a*	QTL(*t*)				4.28*	4.40*	4.87*	4.14*		
		QTL(*t|t* − 1)									
	*d*	QTL(*t*)				4.62*	4.45*				5.50*
		QTL(*t|t* − 1)									5.03**
S_3_	*a*	QTL(*t*)							4.97*	4.74*	6.50*
		QTL(*t|t* − 1)									
S_4_	*a*	QTL(*t*)						6.07**	6.92**	5.11*	5.83*
		QTL(*t|t* − 1)						5.60**			
	*d*	QTL(*t*)		6.22*		6.87**	6.29**	6.83**	9.37**	5.77*	4.87*
		QTL(*t|t* − 1)				3.39*		3.82*			
S_5_	*d*	QTL(*t*)				5.19*					
		QTL(*t|t* − 1)									

SSSL is the abbreviation of the single segment substitution line. S_i_ represented *i*th SSSL. *a* and *d* were additive and dominant effects, respectively, estimated by the mean of $S_{i} - HJX74$(where *i* represented *i*th SSSL that was homozygote or heterozygote). QTL(*t*) and QTL(*t|t* − 1) represent unconditional and conditional QTLs at stage *t*, respectively. $t_{i}$ indicated various developmental stages 7 days apart. Sign “–” indicates descending plant height due to alleles from donors. Superscripts “* and **”Indicate significance at the 5% and 1% levels, respectively.

To understand the interaction mechanism among these QTLs, we first partially aggregated two SSSLs to generate a dual segment substitution line (DSSL) and then carried out a half diallel mating scheme to achieve the various genotypes required to estimate epistasis. Four epistatic components, additive-additive (*aa*), additive-dominance (*ad*), dominance-additive (*da*), and dominance-dominance (*dd*), were estimated according to configurations of genotypes for seven QTL pairs (Table 3). All seven pairs of $S_{i} /S_{j}$ holding significant interaction effects further confirmed the prevalence of epistasis (see $QTL(t)$ in Table 3). Two epistatic components, $d_{1} d_{2}$ (denoted $dd$ of $S_{1} /S_{2}$_,_ similarly hereinafter) and $a_{1} a_{5}$ were detected only at one of stages, and reliability was subject to further verification. The other epistatic components were significant at least at two stages, which indicated the validity of these interactions. Negative epistatic effects were major, while positive epistases mainly appeared at the periods of $t_{8}$ and $t_{9}$. The causes of negative (positive) epistases perhaps were due to positive (negative) additive and dominance effects of QTLs, which will be discussed later. All epistatic effects dynamically changed with developmental stages (see $QTL(t)$ in Table 3).

**Table 3:** Epistatic effects between QTLs estimated on plant height at various developmental stages.

SSSL combination	Epistatic component	QTL	*t*_1_	*t*_2_	*t*_3_	*t*_4_	*t*_5_	*t*_6_	*t*_7_	*t*_8_	*t*_9_
S_1_/S_2_	*ad*	QTL(*t*)								8.21**	8.46**
		QTL(*t|t* − 1)						8.72**			
	*dd*	QTL(*t*)								7.80*	
		QTL(*t|t* − 1)						4.59*		5.96*	− 5.82*
S_1_/S_3_	*aa*	QTL(*t*)				− 9.78**	− 9.01**				10.83**
		QTL(*t|t* − 1)									7.04**
	*dd*	QTL(*t*)									
		QTL(*t|t* − 1)								5.97*	− 5.70*
S_1_/S_4_	*ad*	QTL(*t*)					− 6.45*		− 6.56*		
		QTL(*t|t* − 1)									5.17*
	*da*	QTL(*t*)		6.67*							7.48*
		QTL(*t|t* − 1)		6.10*						6.45*	
	*dd*	QTL(*t*)				− 8.74**	− 11.37**	− 4.35*	− 7.20*		
		QTL(*t|t* − 1)				− 6.37*				5.26*	
S_1_/S_5_	*aa*	QTL(*t*)									8.58**
		QTL(*t|t* − 1)									6.26*
	*da*	QTL(*t*)								8.04**	7.28*
		QTL(*t|t* − 1)								8.37**	
	*dd*	QTL(*t*)									
		QTL(*t|t* − 1)								6.31*	
S_2_/S_3_	*aa*	QTL(*t*)		− 7.76*	− 7.33*	− 8.75**	− 6.66*	− 7.70*			
		QTL(*t|t* − 1)						− 4.50*			
S_2_/S_4_	*aa*	QTL(*t*)			− 7.61*	− 7.76*		− 6.97*			
		QTL(*t|t* − 1)						− 4.12*			
	*ad*	QTL(*t*)		− 9.29**		− 11.17**	− 10.77**	− 8.26**	− 10.25**		
		QTL(*t|t* − 1)				− 5.65*				5.21*	
	*dd*	QTL(*t*)	− 10.96**	− 12.21**	− 9.67**	− 15.07**	− 14.47**	− 8.18*	− 9.97**		− 9.28*
		QTL(*t|t* − 1)	− 10.96**	− 6.19*		− 6.36*					
S_2_/S_5_	*ad*	QTL(*t*)	− 12.02**	− 13.03**	− 9.56**	− 8.77**	− 8.13**	− 7.87*			
		QTL(*t|t* − 1)	− 12.02**	− 6.43*					6.14*		
	*dd*	QTL(*t*)	− 14.16**	− 11.71**	− 9.40**	− 11.66**	− 8.37**				
		QTL(*t|t* − 1)	− 14.16**								

SSSL is the abbreviation of the single segment substitution line. $S_{i}$ represented *i*th SSSL*. aa, ad, da* and *dd* were the abbreviations of epistatic components of additive-additive, additive-dominance, dominance-additive and dominance-dominance, respectively, estimated by the mean of $(D_{ij} + HJX74 - S_{i} - S_{j} )$ (where $D_{ij} , \, S_{i} , \, S_{j}$ indicated dual segment and its two single segment materials, respectively, which might be homozygotes or heterozygotes). QTL(*t*) and QTL(*t|t* − 1) represent unconditional and conditional QTLs at stage *t*, respectively. *t*_*i*_ indicates various developmental stages 7 days apart. Sign “–” indicates descending plant height due to alleles from donors. Superscripts “* and **” indicate significance at the 5% and 1% levels, respectively.

### Conditional QTL mapping on plant height

To acquire the net effect of a given QTL on plant height during a certain period, we carried out conditional QTL mapping. The conditional effects $y_{(t|t - 1)}$, the net effects of phenotypic values at time $t$ given the phenotypic values at time $t - 1$, were first estimated (Supplementary Table 2). Then, conditional QTL effects, the net effects of QTLs from time $t - 1$ to time $t$, were calculated based on the conditional effects $y_{(t|t - 1)}$(see $QTL(t|t - 1)$ in Table 2). Conditional QTLs revealed the quantities and stages of QTL expression. QTL_1_ had two expressions, one was in the stage from $t_{5}$ to $t_{6}$, exhibiting a − 6.57** additive effect, and the other was from $t_{7}$ to $t_{8}$ with a − 3.75* additive effect and a − 4.96** dominant effect. Similarly, QTL_4_ expressed a 3.39* dominant effect from $t_{3}$ to $t_{4}$, a 5.60** additive effect and a 3.82* dominant effect from $t_{5}$ to $t_{6}$, and a QTL_2_ 5.03** dominant effect from $t_{8}$ to $t_{9}$. Although QTL_2_, QTL_3_ and QTL_5_ exhibited significant accumulated effects at certain stages, the concentrated expression stages of these QTLs were not detected due to insufficient large expression quantities. There were no significant expressions to be detected in the early period (from $t_{0}$ to $t_{3}$), and QTL expressions occurred mainly in the middle period (from $t_{3}$ to $t_{6}$) and the late period (from $t_{6}$ to $t_{9}$).

QTL interactions also exhibited different dynamic models (see $QTL(t|t - 1)$ in Table 3). In the early, middle and late periods, there were six, seven and thirteen significant epistatic expressions, respectively. In the three periods, $t_{2} - t_{3}$, $t_{4} - t_{5}$ and $t_{6} - t_{7}$, QTLs hardly expressed. There were 14 significant positive epistatic effects and 12 negative epistatic effects. Mostly, negative expressions appeared in the early period, while positive expressions appeared in the late period. Some epistatic components had significant accumulated effects at certain stages, but their expression periods were not detected due to dispersed expression being insignificant. Conversely, some epistatic components had significant net effects in certain stages but did not detect significant accumulated effects due to reverse expressions. Many epistatic expressions were feeble and failed to be detected, while some large expressions became invisible because of large errors.

### Functional QTL mapping on plant height

Functional QTL mapping is an appropriate method that passes a mathematical equation to describe a biological developmental process with the genetic mapping framework^18^. We first applied the Wang-Lan-Ding model^27^ to fit curves of plant height and to estimate four functional parameters––the optimum time $(t_{0} )$, the growth rate $(r)$, the maximum value $(K)$ and the degradation rate $(c)$(Supplementary Table 3). Then, based on these estimations, we carried out QTL mapping (Table 4). The five SSSLs were found to harbor QTLs with additive and/or dominance to regulate the four parameters. Any one of the SSSLs was associated with at least two functional parameters, for which pleiotropy or close linkage of genes were responsible. S_1_ and S_5_ were involved in all four parameters, and S_2_, S_3_ and S_4_ regulated $t_{0}$ or $K$ and $c$. For the parameter $t_{0}$, QTLs shortened the time of the inflexion point on a curve. For $r$ and $c$, QTLs improved not only the growth rate but also the degradation rate. The impact of QTLs on parameter $K$ differed, enabling the potential of plant height to increase or decrease.

**Table 4:** QTL effects for the four functional parameters on plant height.

SSSL or their combination	Effect	$t_{0}$	$r$	$K$	$c$
S_1_	*a*	− 1.16**	0.16**	− 16.72**	0.27**
	*d*	− 0.46*		− 12.71**	0.25**
S_2_	*d*	− 0.42*			0.18**
S_3_	*a*			6.49*	0.28**
	*d*				0.27**
S_4_	*a*			5.82*	0.22**
	*d*			4.77*	
S_5_	*a*	− 0.46*		− 6.11*	
	*d*	− 0.45*	0.09**	− 4.47*	0.18**
S_1_/S_2_	*aa*	0.80*	− 0.13**	4.19	
	*ad*	1.33**		9.55*	− 0.45**
	*da*			9.62*	− 0.23**
	*dd*			7.95*	− 0.22**
S_1_/S_3_	*aa*	1.77**	− 0.16**	18.38**	− 0.26**
	*ad*	0.79*	− 0.21**		− 0.39**
	*da*				− 0.32**
	*dd*				− 0.52**
S_1_/S_4_	*aa*	0.79*	− 0.18**	8.76*	− 0.25**
	*ad*	0.57*			
	*da*				− 0.47**
	*dd*		− 0.09*		− 0.15*
S_1_/S_5_	*aa*	1.03**	− 0.09*	8.52*	− 0.26**
	*ad*	0.72*	− 0.08*		− 0.19**
	*da*		− 0.09*	16.78**	− 0.23**
	*dd*		− 0.13**	10.39*	− 0.15*
S_2_/S_3_	*aa*	0.59*			− 0.28**
	*ad*	0.58*			− 0.28**
	*da*				− 0.26**
	*dd*				− 0.45**
S_2_/S_4_	*aa*	0.63*			− 0.23**
	*ad*	0.70*			
	*da*	0.60*			− 0.41**
	*dd*	0.91**			
S_2_/S_5_	*aa*			8.66*	0.27**
	*ad*	0.88**			
	*da*	0.97**		10.23*	
	*dd*	1.15**			− 0.17**

SSSL is the abbreviation of the single segment substitution line. $S_{i}$ represents the homozygote or heterozygote *i*th SSSL. The additive effect (*a*) or dominant effect (*d*) of QTLs was estimated by the mean of $(S_{i} - HJX74)$, where HJX74 was the abbreviation of Huajingxian 74. *aa, ad, da* and *dd* represented the additive-additive, additive-dominance, dominance-additive and dominance-dominance epistatic components, respectively, which were estimated by the mean of $(D_{ij} + HJX74 - S_{i} - S_{j} )$ (where $D_{ij} , \, S_{i} , \, S_{j}$ indicated dual segment and its two single segment materials, respectively, which might be homozygotes or heterozygotes). $t_{0} , \, r, \, K$ and *c* were the optimum time, the growth rate, the maximum value and the degradation rate, respectively. Sign “–” indicates descending parameters due to alleles from donors. Superscripts “* and **” indicate significance at the 5% and 1% levels, respectively.

All pairs of S_i_/S_j_ had significant interaction effects, involving one or more parameters by various epistatic components. Interactions between QTL_2_ and QTL_3_, QTL_4_, and QTL_5_ influenced one, two and three parameters, respectively. Epiststatic interactions between QTL_1_ and the other QTLs were associated with all four parameters. Epistases always regulated $t_{0}$ and $K$ positively, while $r$ and $c$ negatively (Table 4). The relationship in which positive (negative) epistasis was always derived from the interaction of negative (positive) QTLs was confirmed once again.

## Discussions

### Dynamic mapping for developmental traits

Most traits of agricultural importance are under the control of an interacting network of genes, which grow and develop through the dynamics of gene expression during the whole growth period^13,14^. Studies via QTL mapping on different kinds of data can provide a wealth of information about the dynamics of QTLs regulating the developmental behavior of quantitative traits^11^. Unconditional QTL mapping of the directly measured phenotypes at various developmental stages can detect the accumulated effects of QTLs before a certain static time point but cannot estimate the expression quantity due to the correlations of phenotypes between two time points^13,14^. Conditional QTL mapping on the indirect estimations of conditional phenotypes can provide net expression of QTLs in a time interval and the stages of QTL expression^16,17^. Functional QTL mapping of the parameters defined in a mathematical function that describes trait variation with biological significance can reveal the QTLs regulating the shape and trajectory of developmental curves^18–22^. In this paper, we carried out a systematic analysis of the dynamics of QTLs regulating the developmental behavior of plant height in rice. We detected that the five SSSLs carried significant additive and/or dominant effects of QTLs on plant height at multiple developmental stages. These QTLs were credible due to their repeatability. Except for QTL_1_, which exhibited negative effects in the middle-late periods, the other QTLs showed enhanced plant height (see QTL(*t*) in Table 2). These QTLs interact with each other to form a genetic network to regulate plant height. The seven pairs of SSSL combinations tested all had one or more significant epistatic components to mostly reduce plant height (see QTL(*t*) in Table 3). QTLs were characterized by temporal expression, selectively appearing significant effects in specific stages of development. The five QTLs turned on mainly in the middle-late periods (see QTL(*t|t* − 1) in Table 2), whereas the seven QTL interactions dispersed in various periods (see QTL(*t|t* − 1) in Table 3). Some expressions were too small to be statistically detected. Plant height varied approximately, followed by the logistic curve of the Wang-Lan-Ding model (Fig. 1), which was determined by the parameters of $t_{0}$, $r$, $K$ and $c$(Wang et al. 1982). These parameters changed the trajectory of the growth curve of plant height, including the inflection point, the growth rate, the peak value and the degradation rate. Our research indicated that the four functional parameters were regulated by the QTLs and the QTL interactions on the five SSSLs, each of which regulated at least two parameters (Table 4).

### Dynamic patterns of QTL expressions

One of the major goals in developmental genetics is to explore gene expression^13,14^. Conditional QTL mapping makes this possible, which can estimate the net expression of QTLs in a certain time interval^16,17^. In theory, the unconditional QTL effect at time point $t$ is the accumulation effect of QTLs from initial time to time $t$, which can be divided into several conditional QTL components, i.e., $QTL_{t} = QTL_{1} + QTL_{2|1} + QTL_{3|2} + \cdots + QTL_{t|t - 1}$. Conditional QTL effects were independent of each other and thus were additive. According to the formula, it is possible to generate follow a few cases at stage $t$, being significant for both $QTL_{t}$ and $QTL_{t|t - 1}$, either $QTL_{t}$ or $QTL_{t|t - 1}$, and neither $QTL_{t}$ nor $QTL_{t|t - 1}$. The relationship between unconditional QTLs and conditional QTLs was discussed in our previous paper^30^ and was well validated by the results estimated in this paper. The correlation coefficient between $QTL$ effects at the final stage $t_{9}$ and the sum of all conditional QTL effects before $t_{9}$ reached 0.9379** in the previous paper^30^ and 0.7208^**^ in this paper (data not shown). Only a series of conditional QTLs truly reflected the expression periods and quantities of a QTL throughout the whole developmental stage. Conditional QTL mapping has widely been applied to reveal dynamic gene patterns for developmental traits^8,9,15,16,23,30,31^. There were four representative patterns for the genetic control of growth trajectories, permanent QTLs, early QTLs, late QTLs and inverse QTLs^21^. This knowledge derived from the accumulated effects of QTLs, QTLs being permanent, early, late and inverse when one genotype was better than the other in entire growth process, at early stages, at late stages and one genotype showed inverse effects with the other since a particular stage, respectively. However, the accumulated effects of QTLs could not reflect the expression stages and quantities of QTLs. This paper indicated that QTLs for plant height expressed all additive, dominant and epistatic effects according to the temporal expression pattern (see QTL(*t|t* − 1) in Tables 2 and 3). QTLs and their interactions expressed significant effects only in one or more periods and sometimes even had no significant expression periods while remaining silent all the time. Permanent expression of QTLs was rare. QTL_1_ and QTL_2_ were expressed mainly in the late period, QTL_4_ was expressed in the middle period, and QTL_3_ and QTL_5_ were not significantly expressed (see QTL(*t|t* − 1) in Table 2). Similarly, QTL_1_/QTL_2_, QTL_1_/QTL_3_, QTL_1_/QTL_5_ and QTL_2_/QTL_3_ were expressed mainly since period $t_{5}$, QTL_1_/QTL_4_ and QTL_2_/QTL_4_ were dispersed in various periods, and QTL_2_/QTL_5_ had inverse effects between the early period and the late period (see QTL(*t|t* − 1) in Table 3). In fact, QTLs and their interactions expressed net effects in various stages, just some of which reached the levels of significance statistically. Small expressions of QTLs were considered as no expressing or experimental error.

### QTLs regulated developmental trajectories of temporal traits

**Figure 3:**
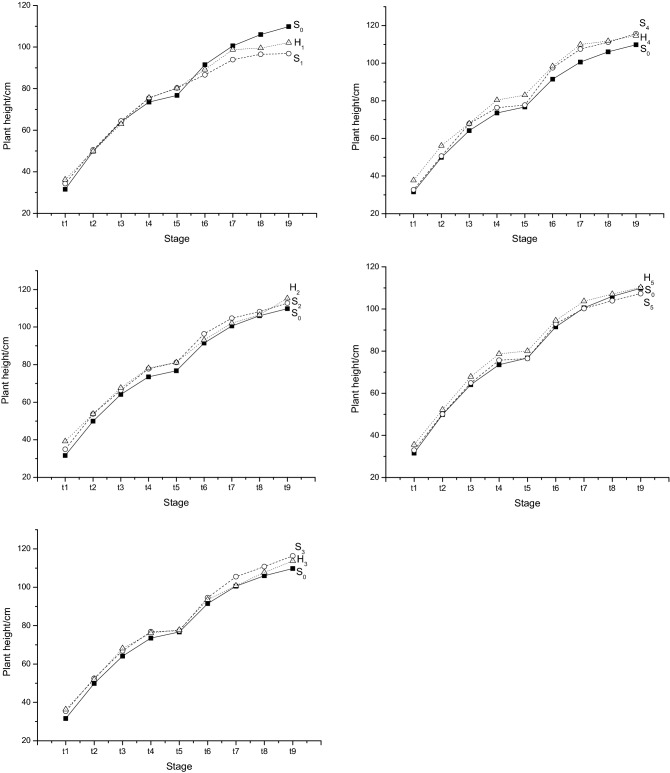
Different trajectories corresponding to different genotypes at a given QTL. $S_{0}$ represents the genotype (*aa*) of HJX74, while $S_{i}$ and $H_{i}$ indicate the genotypes (*AA* and *Aa*) of the *i*th single segment substitution line*. ti* indicates various developmental stages, and the difference at 7 d. Unit of plant height was in cm.

Developmental theory considers that if different genotypes at a given QTL correspond to different developmental trajectories, the QTL must affect the differentiation of this trait^21^. Therefore, by estimating the functional parameters that define the trait curve of each QTL genotype and testing the differences in these parameters among genotypes, one can determine whether a QTL affects the formation and expression of a trait during development. In the Wang-Lan-Ding model, there were four parameters to regulate the growth curves of developmental traits, which might change the inflexion point ($t_{0}$), the upper limit ($K$), the rise speed ($r$) and the descent speed ($c$) of curves^27^. In this paper, genotypes of five SSSLs differed from that of HJX74 at a given QTL (Fig. 3), implying that a putative QTL existed on each of the SSSLs. Both unconditional QTL mapping and conditional QTL mapping confirmed the existence of QTLs (Table 2). How did these QTLs affect the development of plant height? Functional QTL mapping based on the estimations of the four parameters indicated that QTL_1_ and QTL_5_ regulated all four parameters by additive and/or dominant effects, and the other three QTLs influenced two of them, $t_{0}$ or $K$ and $c$, respectively. These QTLs brought the inflexion point ahead of schedule and accelerated the growth and degradation of plant height. QTL_1_ and QTL_5_ made the maximum plant height shorter, while QTL_3_ and QTL_4_ had higher plant heights (Table 4). Similarly, the interactions among these QTLs also influenced the four parameters, which always positively regulated $t_{0}$ and $K$, while $r$ and $c$ negatively regulated by various epistatic components (Table 4).

### Impact of epistasis on plant height

In multiple gene systems, gene interactions are inevitable except for gene additives, which include allelic interactions (dominance) and nonallelic interactions (epistasis)^32,33^. For statistical purposes, genotypic effects can be divided into single locus effects (additive or dominance) and interaction effects among segregating loci (epistasis)^34^. Thus, epistasis is an important genetic component and a plausible feature of the genetic architecture of quantitative traits. Mapping epistatic interactions is challenging experimentally, statistically and computationally, which requires large sample sizes, severe penalties and a large number of tests^35^. QTL mapping studies using primary mapping populations such as F_2_, BC, DHLs and RILs cannot clearly support the existence of specific interactions among QTLs because of the limitation of inconsistent genetic backgrounds among individuals or lines^26^. SSSLs or NILs (near isogenic lines) have huge advantages for QTL identification in general and characterization of epistasis in particular^26,36,37^. On the one hand, target QTLs can be detected by testing the difference between any one of the SSSLs and the receptor parent; on the other hand, pyramiding of SSSLs enables estimation of epistatic effects^38–44^. In this paper, we first detected five SSSLs that carry significant additive and/or dominant effects of QTLs on plant height (Table 2), and then four components of epistases were estimated via analysis of pyramiding effects derived from two SSSLs (Table 3). The information is reliable due to the repeated emergence of putative QTLs and their interactions at multiple stages of development. All seven pairs of SSSLs were tested with two or more significant effects of four epistatic components, further confirming the prevalence of epistasis (Table 3). Epistasis may be brought about by modification of gene function due to alterations in the signal-transducing pathway. Epistatic genes are more deleterious in combination than separately, which are often accompanied by inverse epistatic interactions as homeostatic (that is, canalizing) mechanisms^35^. This role of epistasis was first observed by Eshed and Zamir^37^ when they found less-than-additive interactions between QTLs in tomato. We also confirmed an inverse epistastic role of yield traits in rice, negative epistasis being derived mainly from interactions between positive QTLs, while positive epistasis from negative QTLs^30,43^. In this paper, most QTLs were detected with positive additive and/or dominance; thus, the estimations of epistatic components were mainly negative. Positive epistasis occurred only after the stage of $t_{6}$ since negative QTLs appeared (Tables 2 and 3). The property of epistasis was stipulated by the calculated formula $e_{ij} = g_{ij} - a_{i} - a_{j}$, where $e, \, g, \, a$ indicated epistatic, genotypic and two of single locus effects, respectively, and $i, \, j$ represented two loci. Because the value of $e_{ij}$ is inversely proportional to the sum of single locus effects, it is more likely to gain negative (positive) epistasis when there are positive (negative) single locus effects. Additionally, one QTL always interacted with multiple other QTLs, forming a genetic network. In this paper, five QTLs were detected to interact with each other with one or more significant epistatic components (Table 3). Of the seven combinations of SSSL sets, QTL_1_ and QTL_2_ interacted with the other four QTLs, while QTL_3_, QTL_4_, and QTL_5_ interacted with at least the other two QTLs. Five QTLs had various interaction magnitudes, displaying different epistatic intensities. QTL_2_ and QTL_4_ seemed to have larger average epistatic effects and greater interoperability than the other QTLs (Supplementary Table 4). Of the four epistatic components, the average estimation of *dd* was seemingly larger than those of the others (Supplementary Table 4). Knowledge of epistatic interactions will improve our understanding of genetic networks and mechanisms that underlie genetic homeostasis and improve predictions of responses to artificial pyramiding breeding for quantitative traits in agricultural crop species. In the future, we must assess the effects of pairwise and higher-order epistatic interactions between polymorphic DNA variants on molecular interaction networks and, in turn, evaluate their effects on organismal phenotypes to understand the mechanistic basis of epistasis^35^. Only then will we be able to go beyond describing the phenomenon of epistasis to predicting and testing its consequences for genetic systems.

## Supplementary Information

Supplementary Information.
