# Supplementary material for: Dynamic analysis of QTLs on plant height with single segment substitution lines in rice
Source: Sci Rep. 2022 Mar 31;12:5465. doi: 10.1038/s41598-022-09536-8 (PMC8971505; doi:10.1038/s41598-022-09536-8)
Supplement: Supplementary file 1 — Supplementary Information. [file 41598_2022_9536_MOESM1_ESM.docx]

Supplementary Table 1 Analysis of variance for plant height at each of stages. MS_t_ and MS_e_ were mean square due to different genotypes and residual error, with the freedom degree of 38 and 78, respectively. t_1_~t_9_ represented the measured stages for plant height, 7 days apart.

| Item | t_1_ | t_2_ | t_3_ | t_4_ | t_5_ | t_6_ | t_7_ | t_8_ | t_9_ |
| --- | --- | --- | --- | --- | --- | --- | --- | --- | --- |
| MS_t_ | 16.86 | 21.64 | 17.72 | 21.94 | 21.1 | 27.52 | 50.92 | 51.85 | 81.06 |
| MS_e_ | 26.32 | 14.03 | 9.96 | 12.23 | 13.62 | 9.23 | 8.48 | 10.2 | 12.78 |
| F-value | 0.641 | 1.542 | 1.779 | 1.794 | 1.549 | 2.982 | 6.005 | 5.083 | 6.343 |
| P-value | 0.948 | 0.042 | 0.011 | 0.010 | 0.041 | 0.000 | 0.000 | 0.000 | 0.000 |

Supplementary Table 2 Estimations of conditional effects for various genotypes at nine measured stages. HJX74 was the abbreviation of Huajingxin 74. S_i_ and D_i_ represented the homozygote and heterozygote of *i*th SSSL, respectively. Combinations between S_i_ or D_i_ and S_j_ or D_j_ were pyramiding materials of two single segment materials..$y_{(t|t-1)}$indicated conditional variable, the net effect from times $(t-1)$ to $(t)$ which was estimated by the phenotypic values at time$(t)$given those at time $(t-1)$.

| Material | $y_{(t1\vert t0)}$ | $y_{(t2\vert t1)}$ | $y_{(t3\vert t2)}$ | $y_{(t4\vert t3)}$ | $y_{(t5\vert t4)}$ | $y_{(t6\vert t5)}$ | $y_{(t7\vert t6)}$ | $y_{(t8\vert t7)}$ | $y_{(t9\vert t8)}$ |
| --- | --- | --- | --- | --- | --- | --- | --- | --- | --- |
| HJX74 | -2.62 | 0.60 | -0.56 | -1.44 | 0.20 | -0.26 | -0.03 | 0.78 | -0.15 |
| S_1_ | 0.14 | -0.26 | -0.67 | 0.16 | 2.07 | -6.83 | -1.96 | -2.97 | -3.03 |
| S_2_ | 0.74 | 2.61 | -0.93 | 0.82 | 0.71 | 2.50 | -0.61 | -0.65 | 0.47 |
| S_3_ | 1.20 | 1.13 | 0.44 | -0.64 | -2.21 | 2.56 | 1.93 | 1.20 | 1.32 |
| S_4_ | -1.51 | 0.67 | 2.68 | -1.92 | -1.42 | 5.34 | 1.00 | -0.12 | 0.26 |
| S_5_ | -1.26 | 0.04 | 0.11 | 0.02 | -1.97 | 1.29 | -1.75 | -1.03 | -0.44 |
| D_1_ | 2.02 | -1.98 | -1.69 | 1.68 | 1.70 | -4.16 | 0.35 | -4.18 | -0.92 |
| D_2_ | 5.02 | 0.30 | 0.32 | 0.01 | 0.45 | -0.77 | -0.03 | -0.15 | 4.88 |
| D_3_ | 2.18 | -0.02 | 2.16 | -2.38 | -1.25 | 1.28 | -1.73 | 2.26 | 1.93 |
| D_4_ | 3.51 | 3.46 | -0.92 | 1.95 | 0.24 | 3.56 | 2.72 | -1.60 | -1.40 |
| D_5_ | 1.43 | 0.67 | 1.62 | 0.39 | -1.23 | 1.23 | 0.16 | -0.91 | -0.86 |
| S_1_/S_2_ | -2.16 | -0.29 | -0.52 | 0.38 | 0.47 | -4.07 | -1.67 | 0.27 | -4.75 |
| S_1_/S_3_ | -1.97 | -4.52 | -0.49 | -3.15 | -0.46 | -1.97 | 0.66 | 2.38 | 5.49 |
| S_1_/S_4_ | -1.13 | -0.81 | -1.49 | 0.69 | -0.59 | -1.82 | -0.82 | -1.87 | 1.53 |
| S_1_/S_5_ | -2.41 | 0.14 | -3.09 | 1.68 | -1.19 | -4.69 | -2.93 | -2.04 | 2.94 |
| S_2_/S_3_ | -1.32 | -1.38 | -1.98 | -0.53 | -0.41 | 0.81 | 2.87 | 0.08 | 0.67 |
| S_2_/S_4_ | -2.51 | -0.23 | -1.71 | -0.56 | 0.20 | 3.98 | 3.57 | -0.06 | 1.80 |
| S_2_/S_5_ | 1.70 | 1.56 | 1.99 | -0.26 | -0.06 | 2.82 | 0.22 | -0.13 | -0.48 |
| S_1_/D_2_ | 2.04 | -3.08 | -1.04 | 1.15 | 0.65 | 1.38 | -2.50 | -0.04 | 1.77 |
| S_1_/D_3_ | 2.32 | -0.36 | 0.18 | 0.89 | 1.68 | -5.19 | -1.12 | 0.22 | -3.37 |
| S_1_/D_4_ | 0.42 | 0.83 | 1.16 | 1.31 | -1.32 | -3.34 | -2.46 | -2.61 | 0.89 |
| S_1_/D_5_ | -0.12 | 1.52 | -1.31 | 1.35 | 0.94 | -5.14 | -3.11 | -0.82 | -1.97 |
| S_2_/D_1_ | -1.06 | 1.77 | -1.04 | 2.36 | 2.38 | -1.54 | 1.00 | 0.70 | -3.58 |
| S_2_/D_3_ | 3.89 | 1.04 | 1.06 | -0.84 | 0.53 | 3.95 | -0.10 | 2.94 | -0.03 |
| S_2_/D_4_ | -2.55 | 1.36 | -1.12 | -1.44 | 0.14 | 3.22 | -0.10 | 2.19 | -0.61 |
| S_2_/D_5_ | -7.23 | -3.75 | 0.54 | 2.49 | -0.88 | 0.02 | 5.72 | -1.77 | -0.62 |
| S_3_/D_1_ | -1.03 | -1.96 | 0.33 | -1.36 | 1.69 | -1.54 | 0.70 | -1.05 | 2.62 |
| S_3_/D_2_ | -1.43 | 1.32 | 2.55 | -2.78 | -0.81 | 2.38 | 2.08 | -0.32 | 1.65 |
| S_4_/D_1_ | 4.16 | 4.19 | 1.71 | 1.58 | -2.52 | 0.59 | 0.89 | 1.36 | 0.67 |
| S_4_/D_2_ | -0.13 | -0.04 | -0.70 | -1.26 | -0.70 | 4.32 | 3.09 | 1.07 | 0.38 |
| S_5_/D_1_ | 0.47 | 0.29 | -1.26 | 0.89 | 1.89 | -4.91 | 0.10 | 2.38 | -2.46 |
| S_5_/D_2_ | -2.74 | 1.47 | 1.34 | -1.18 | 1.58 | 4.30 | -0.61 | -0.09 | 1.95 |
| D_1_/D_2_ | 0.71 | -1.36 | 1.87 | 1.14 | 0.26 | -0.08 | -0.35 | 0.85 | -1.71 |
| D_1_/D_3_ | 1.98 | -0.27 | -0.67 | 1.81 | 0.13 | -1.48 | -4.30 | 3.28 | -4.54 |
| D_1_/D_4_ | 0.44 | -1.05 | -0.50 | -1.30 | -1.68 | 0.76 | 0.12 | -1.30 | 1.55 |
| D_1_/D_5_ | 1.96 | 0.18 | 0.87 | 2.34 | 1.98 | -3.08 | -3.53 | 0.43 | -3.17 |
| D_2_/D_3_ | 1.73 | 2.63 | 1.11 | 0.29 | -1.99 | 1.17 | 0.85 | 0.07 | 2.02 |
| D_2_/D_4_ | 0.18 | -3.04 | -1.42 | -2.96 | -0.27 | 1.81 | 0.68 | 2.09 | -1.35 |
| D_2_/D_5_ | -5.09 | -3.56 | 1.05 | -1.35 | 1.26 | 1.43 | 0.85 | -0.73 | 0.77 |

Supplementary Table 3 Estimations of functional parameters in the Wang-Lan-Ding model on plant height. $t_{0},\text{ }r,\text{ }K$ and $c$ were the optimum time , the growth rate , the maximum value and the degradation rate, respectively. HJX74 was the abbreviation of Huajingxin 74. S_i_ and D_i_ represented the homozygote and heterozygote of *i*th SSSL, respectively. Combinations between S_i_ or D_i_ and S_j_ or D_j_ were pyramiding materials of two single segment materials.

| Material | $t_{0}$ | $r$ | $K$ | $c$ |
| --- | --- | --- | --- | --- |
| HJX74 | 2.84 | 0.39 | 119.81 | 0.03 |
| S_1_ | 1.68 | 0.55 | 103.09 | 0.30 |
| S_2_ | 2.56 | 0.42 | 120.31 | 0.03 |
| S_3_ | 2.74 | 0.36 | 126.30 | 0.31 |
| S_4_ | 2.88 | 0.42 | 125.63 | 0.25 |
| S_5_ | 2.38 | 0.44 | 113.70 | 0.03 |
| D_1_ | 2.38 | 0.43 | 107.10 | 0.28 |
| D_2_ | 2.42 | 0.37 | 121.34 | 0.21 |
| D_3_ | 2.45 | 0.40 | 120.37 | 0.30 |
| D_4_ | 2.50 | 0.40 | 124.58 | 0.03 |
| D_5_ | 2.39 | 0.48 | 115.34 | 0.21 |
| S_1_/S_2_ | 2.20 | 0.45 | 107.78 | 0.25 |
| S_1_/S_3_ | 3.35 | 0.36 | 127.96 | 0.33 |
| S_1_/S_4_ | 2.51 | 0.40 | 117.67 | 0.28 |
| S_1_/S_5_ | 2.26 | 0.50 | 105.50 | 0.04 |
| S_2_/S_3_ | 3.04 | 0.39 | 122.77 | 0.03 |
| S_2_/S_4_ | 3.22 | 0.40 | 127.74 | 0.02 |
| S_2_/S_5_ | 2.49 | 0.40 | 122.86 | 0.29 |
| S_1_/D_2_ | 2.60 | 0.49 | 114.17 | 0.03 |
| S_1_/D_3_ | 2.08 | 0.35 | 110.78 | 0.18 |
| S_1_/D_4_ | 1.92 | 0.59 | 103.62 | 0.29 |
| S_1_/D_5_ | 1.95 | 0.55 | 105.10 | 0.29 |
| S_2_/D_1_ | 2.50 | 0.45 | 117.22 | 0.05 |
| S_2_/D_3_ | 2.74 | 0.40 | 126.22 | 0.02 |
| S_2_/D_4_ | 2.91 | 0.40 | 123.86 | 0.04 |
| S_2_/D_5_ | 2.98 | 0.48 | 118.90 | 0.29 |
| S_3_/D_1_ | 2.75 | 0.44 | 116.52 | 0.25 |
| S_3_/D_2_ | 2.87 | 0.40 | 125.21 | 0.24 |
| S_4_/D_1_ | 2.14 | 0.50 | 119.13 | 0.04 |
| S_4_/D_2_ | 3.05 | 0.40 | 127.43 | 0.02 |
| S_5_/D_1_ | 2.21 | 0.39 | 117.77 | 0.04 |
| S_5_/D_2_ | 2.93 | 0.40 | 125.47 | 0.22 |
| D_1_/D_2_ | 2.34 | 0.44 | 116.58 | 0.24 |
| D_1_/D_3_ | 2.08 | 0.47 | 107.29 | 0.04 |
| D_1_/D_4_ | 2.53 | 0.36 | 116.38 | 0.13 |
| D_1_/D_5_ | 2.09 | 0.39 | 113.02 | 0.31 |
| D_2_/D_3_ | 2.32 | 0.39 | 117.94 | 0.03 |
| D_2_/D_4_ | 2.99 | 0.40 | 125.52 | 0.26 |
| D_2_/D_5_ | 3.11 | 0.46 | 119.51 | 0.23 |

Supplementary Table 4 Average epistatic effects for combinations of each SSSL and for each component. SSSL was the abbreviation of single segment substitution line. S_i_ represented the code of *i*th SSSL*.* *t_i_* indicated various developmental stages, the difference of 7d. *aa, ad, da* and *dd* were additive-additive, additive-dominance, dominance-additive and dominance-dominance epistasis respectively. Sign “–” meant to descend plant height due to the alleles from donors. Superscripts “^*^ and ^**^” indicated the significance at 5% and 1% level, respectively.

| Item | t_1_ | t_2_ | t_3_ | t_4_ | t_5_ | t_6_ | t_7_ | t_8_ | t_9_ |
| --- | --- | --- | --- | --- | --- | --- | --- | --- | --- |
| S_1_/- | -4.83^**^ | -2.34 | -1.99 | -3.19^*^ | -3.48^*^ | -0.82 | -1.44 | 3.37^*^ | 4.12^*^ |
| S_2_/- | -7.46^**^ | -5.76^**^ | -4.51^*^ | -6.16^**^ | -5.32^**^ | -2.59 | -1.34 | 1.38 | -1.10 |
| S_3_/- | -5.77^**^ | -3.73^*^ | -3.39^*^ | -4.36^**^ | -3.22^*^ | -1.64 | -0.96 | 1.16 | -0.34 |
| S_4_/- | -5.74^**^ | -4.63^**^ | -4.35^**^ | -6.53^**^ | -7.23^**^ | -4.67^**^ | -5.24^**^ | -0.82 | -0.19 |
| S_5_/- | -6.37^**^ | -3.69^*^ | -3.17^*^ | -4.42^**^ | -2.76 | -1.68 | -0.71 | 2.78 | 2.71 |
| *aa* | -3.99^*^ | -4.26^**^ | -4.61^**^ | -5.65^**^ | -5.11^**^ | -3.62^*^ | -2.12 | 0.70 | 2.97 |
| *ad* | -5.95^**^ | -5.18^**^ | -4.21^*^ | -5.00^**^ | -4.94^**^ | -2.57 | -1.94 | 1.13 | 1.43 |
| *da* | -5.80^**^ | -1.48 | -1.16 | -3.12^*^ | -1.75 | -0.86 | -0.30 | 3.51^*^ | 1.86 |
| *dd* | -8.38^**^ | -5.21^**^ | -3.95^*^ | -5.97^**^ | -5.80^**^ | -2.07 | -3.39^*^ | 0.95 | -2.10 |
